# The Role of Dietary Fatty Acids in Modulating Blue Crab (*Callinectes sapidus*) Physiology, Reproduction, and Quality Traits in Captivity

**DOI:** 10.3390/ani14223304

**Published:** 2024-11-17

**Authors:** Federico Conti, Lina Fernanda Pulido-Rodriguez, Giulia Chemello, Nico Cattaneo, Mattia Resente, Giuliana Parisi, Ike Olivotto, Matteo Zarantoniello

**Affiliations:** 1 Department of Life and Environmental Sciences, Università Politecnica delle Marche, 60131 Ancona, Italy; f.conti@pm.univpm.it (F.C.); mattiaresente19@gmail.com (M.R.);; 2 Department of Agriculture, Food, Environment and Forestry, University of Florence, 50144 Firenze, Italy; linafernanda.pulidorodriguez@unifi.it (L.F.P.-R.); giuliana.parisi@unifi.it (G.P.)

**Keywords:** growth performance, PUFA, hepatopancreas, animal welfare

## Abstract

The invasive blue crab is challenging the Mediterranean basin, progressively declining local populations. This reflects a lower prey availability and suitability of dietary nutrients (mainly n-3 polyunsaturated fatty acids, PUFA). The present study aimed to challenge blue crab males and females with a feed source low in n-3 PUFA with respect to one showing a proper fatty acid profile and to investigate the responses in terms of growth, welfare, lipid characterization of target tissues, and reproductive status. Blue crabs were divided into three groups as follows: (i) Marine: crabs fed sardinella (*Sardinella aurita*) fillet for 60 days; (ii) Mix: crabs fed bovine heart for the first 40 days and sardinella fillet for the following 20 days; and (iii) Terrestrial: crabs fed bovine heart for 60 days. The diet did not alter the health status but reflected the fatty acid profile of muscle and ovary of the blue crabs. In each group, males and females showed a proper hepatopancreas structure, with comparable levels of lipid reserves. This properly supported gonad maturation in both sexes. However, males and females from the group fed the terrestrial diet were characterized by reduced body weight, revealing that blue crabs prioritize reproductive investment rather than growth by directing crucial nutrients to reproductive organs when a suboptimal diet is available.

## Simple Summary

The rapid expansion of blue crab in the Mediterranean Sea represents a severe threat for both human activities and biodiversity. It is a highly invasive species, with high fecundity, and able to tolerate a wide range of temperatures and salinities as well as to afflict the trophic net of local benthic ecosystems at multiple levels. Despite being omnivorous, the progressively lower prey abundance and variability can lead to a consequent lack of crucial dietary nutrients (mainly lipids) that may alter the population dynamics over time. The present study compared the effects on blue crabs of two distinct diets (a marine and a terrestrial one) with different fatty acid profiles over a 60-day feeding trial. The results highlighted how both males and females were able to face the dietary lack of crucial nutrients by preferentially investing them in reproductive performances at the expense of growth.

## 1. Introduction

Besides habitat loss and overfishing, the introduction of non-indigenous species is one of the most critical stressors for marine ecosystems, particularly affecting coastal areas [1,2]. In the last few decades, the Mediterranean Sea has been affected by a dramatic increase of alien flora and fauna [3,4], with crustaceans (mainly brachyurans) that are addressed as emblematic taxa of this process [5,6]. Particularly, the blue crab, *Callinectes sapidus* (Brachyura, Portunidae), was accidentally introduced by ballast waters from the western Atlantic Ocean to the Atlantic coast of France, and it has progressively spread in the Mediterranean Sea since 1947 [7,8,9]. The expansion affected the western Mediterranean Sea, the Portuguese Atlantic waters, the Ionian-Adriatic area, and the Levantine basin in which, to date, the blue crab is almost ubiquitous [10]. This rapid expansion represents a severe threat for fisheries, by damaging the nets and by decreasing the availability of some fish, molluscs or crustacean species of commercial interest, with negative economic and social consequences, as well as for biodiversity and the functioning of local benthic ecosystems [10,11,12]. In fact, the blue crab is considered among the 100 worst invasive alien species in the Mediterranean Sea due to the match of its ecological and trophic needs with the environmental and ecosystem features of this area [13,14]. Despite the absence of an official program to manage the invasion in the Mediterranean Sea, recently the blue crab fishery has been adopted as a population control measure in certain Mediterranean regions, possibly reducing the number of specimens in a certain area and generating an alternative economical income for fishermen [15]. However, to implement such measures, research is needed to determine the temporal population size distribution, growth periods, and sexual maturity, considering local environmental conditions, which makes this option susceptible to geographical variations [16].

The blue crab is an euryhaline and eurythermal species that is able to colonize coastal habitats like estuaries and lagoons with freshwater inputs [9,17]. Different salinity levels are usually experienced by this species in order to complete its life cycle [10]. In fact, blue crab mating mostly occurs in low-salinity coastal environments, during which males transfer, through the pleopods, the seminal fluid containing the spermatophores to females that store it in the seminal receptacles for several months [18]. In the first autumn, after recovering from molting and mating, females start migrating and undergo oocyte development and growth; finally, in late spring, they reach high-salinity areas where full-grown oocytes are fertilized with the previously stored sperm and subsequently hatch in planktonic zoea larvae [18,19]. The blue crab’s high fecundity can be attributed to the small size of the individual eggs, the large internal space available for developing ovaries, and the extensive abdominal area suitable for external brooding [20,21]. A single female typically produces more than 2 million eggs in its first brood [22], an amount that decreases in the following reproductive events but that is higher compared with most other crab species [23]. In winter, when the water temperature drops below 10 °C, blue crabs become inactive, leading to the absence of reproductive events and to high mortality rates [24]. However, the ongoing warming of the Mediterranean basin, especially in the Adriatic/Ionian area [25,26] has increased the survival and maturation rates of overwintering populations, positively affecting their fitness. This, further improved their expansion which, in turn, is not contained due to the absence of predators, parasites, or pathogens in these areas [10,27].

The widespread presence of the blue crab in the Mediterranean Sea poses a risk to the native communities due to its aggressive predatory behavior and the ability to exploit the food chain of the benthic ecosystem at multiple levels [28,29]. In fact, the blue crab is a generalist omnivore [30], able to localize the prey through chemical stimuli [31] as well as to discriminate them in relation to their size, reducing the time spent for manipulation and to their preferences [32]. It can rely on a wide range of feed sources depending on their availability, including bivalves, fish, crustaceans, and, to a lesser extent, those of plant origin [1]. This opportunistic and aggressive feeding habit leads to adverse interactions with native crustacean populations [33,34], including predation and competition with the Mediterranean green crab (*Carcinus aeustuarii*) [35] and the caramote prawn (*Penaeus kerathurus*) [36] and the predatory extirpation of the Japanese common clam (*Ruditapes philippinarum*) [37,38].

The specific diet of the blue crab can also vary according to their sex, with males relying on a more constant diet throughout the year due to a restricted living area, while females switch their diet according to migration in offshore waters and higher sensitivity to low water temperatures compared to males [39,40]. During winter, food availability is lower, leading to dietary shifts that can cause population decline by affecting survival, reproductive performance, and offspring production [41].

The strong pressure of the invasive blue crab on the benthic ecosystem of the Mediterranean Sea reduces both the availability and variability of common prey for this species. Despite its generalist omnivorous nature, the consequent trophic shift to suboptimal food sources and the long-term lack of crucial dietary nutrients may alter the population dynamics of this species [42]. Particularly, dietary lipids are crucial for providing a source of energy and regulating many developmental and physiological processes in marine crustaceans [43]. In fact, dietary cholesterol is the precursor of ecdysteroids which, after binding the heterodimeric receptor (in crustaceans composed of ecdysone receptor—EcR—and retinoid X receptor—RXR), initiates a transcriptional cascade of responsive genes in target tissues, regulating growth, molting, and reproduction [44,45]. In addition, for these processes, long-chain polyunsaturated fatty acids (PUFA), particularly those of the n-3 series, are considered crucial, specifically because, in these animals, the synthesis of eicosapentaenoic (C20:5n-3; EPA), docosahexaenoic (C22:6n-3; DHA), and arachidonic acid (C20:4n-6; ARA) acids from their respective precursors is severely scarce. Therefore, these fatty acids are considered as essential nutrients that need to be ensured by the diet [46]. Accordingly, dietary deficiencies or imbalances in these fatty acids may result in reduced survival, poor growth, prolonged intermolt periods, and impaired reproductive performance [47,48,49,50]. 

In this context, the aim of the present study was to investigate the effects on the main processes governing the blue crab population dynamics (growth and reproduction) of a feeding regimen characterized by an unbalanced profile of crucial fatty acids. For that reason, blue crab males and females were challenged with bovine heart as a feed source characterized by a suboptimal n-3 PUFA profile, due to its terrestrial origin [51]. This dietary source was selected because it is a cheap and poorly used food source for human nutrition, which can be revalorized as feed. In comparison, the fish sardinella (*Sardinella aurita*) was selected as a marine dietary source, particularly rich in n-3 PUFA, that could represent a potential realistic feed to be used in view of blue crab farming due to its wide availability in the Adriatic area and because it represents a fishery by-product that could be revalorized. Particularly, blue crab farming may serve to increase the Adriatic population, which is usually smaller than the native Atlantic one, thus having a very lower commercial value.

The selected diets were provided to blue crab males and females over a 60-day feeding trial in controlled conditions to investigate the physiological responses in terms of growth, welfare, lipid characterization in target tissue, quality traits, and reproductive status. For that reason, particular emphasis was given to: (i) the hepatopancreas, as the largest organ in the digestive tract of Decapoda, involved in absorption, metabolization, and storage of nutrients, as well as in the stress response [52]; (ii) the gonads since their status is dependent on the nutrients received from the hepatopancreas reserves and, thus, from nutrition [53]; and (iii) the lipid content and fatty acid profile of breast muscle and ovary since both are subjected to the nutritional composition of the administered diet and can thus affect both quality traits and the reproductive success of this species [47,54].

## 2. Materials and Methods

### 2.1. Ethics

Optimal rearing conditions were maintained throughout the study, and all efforts were made to minimize animal suffering. The feeding trial was carried out according to the European Directive 2010/63/EU of the European Parliament and of the Council of the European Union on the protection of animals used for scientific purposes. Because the blue crab is an invertebrate, no specific permit is required under the current law.

### 2.2. Experimental Diets

Two experimental diets were tested in the present study. The marine diet consisted of sardinella (*Sardinella aurita*) fillet, provided by a local fisherman. The challenge diet (terrestrial origin) consisted of bovine heart, purchased in a local market (Ancona, Italy) and selected for its very low price. The lipid content and the fatty acid profile of each diet is reported in Table 1.

### 2.3. Experimental Design

One hundred and eighty blue crabs (initial body weight 80.7 ± 1.6 g), in the intermolt phase (ensured by the carapace rigidity and the absence typical molting features [55]), provided by fishermen of Goro (Ferrara, Italy) in September, were subjected to a 2-week acclimation in a single 2000 L tank equipped with mechanical, biological, and UV filtration (Panaque s.r.l., Viterbo, Italy), with the following water parameters: temperature, 21 °C; salinity 35‰; ammonia and nitrite < 0.05 mg/L; nitrate < 10 mg/L [56]. After the acclimation period, blue crabs were randomly divided into three experimental groups (in triplicate), according to the dietary treatments, as follows: (i) Mar group: blue crabs fed sardinella (*Sardinella aurita*) fillet for the whole trial (60 days); (ii) Mix group: blue crabs fed bovine heart for the first 40 days and sardinella (*Sardinella aurita*) fillet for the remaining 20 days; and (iii) Ter group: blue crabs fed bovine heart for the whole trial (60 days).

Each experimental group (60 blue crabs per group; 20 blue crabs per tank, 10 males and 10 females per tank) consisted of three 500 L tanks singularly equipped with mechanical and biological filtration (Panaque). Throughout the whole trial, blue crabs were subjected to a natural photoperiod (12 light/12 dark), and water parameters were the same reported for the acclimation period (maintained constant for the whole trial). In each tank, fine sand was placed on the bottom, and several rocks and PVC pipes were included in the tank to provide shelters to reduce aggression. Each diet was provided at 2% body weight, divided into one ration in the morning and one in the afternoon, according to the fact that after 1 h of feed administration, all the feed provided was completely ingested by the crabs. The daily ration was adjusted every two weeks by weighing all the specimens from each tank. A visual inspection of each tank to check the eventual presence of dead specimens was conducted daily.

At the end of the feeding trial (60 days), all the crabs in each tank were collected and individually measured with a caliper (for carapace length and width; precision 1 mm) and weighed (digital balance, precision 0.1 g). Crabs were anaesthetized in ice (−20 °C for 15 min [57]), and the dorsal carapace was removed to sample the organs.

Whole gonads and the hepatopancreas were removed and weighed to calculate gonadosomatic (GSI) and hepatosomatic (HSI) indexes, as follows:

GSI = (weight of the gonad/final body weight) × 100,(1)

HSI = (weight of the hepatopancreas/final body weight) × 100(2)

Finally, the hepatopancreas, gonads, pericardial organ, and breast muscles were sampled and properly stored for further analyses, as reported in the dedicated sections.

### 2.4. Chemical Analyses

The following samples were collected for the chemical analysis: (i) whole breast muscle from 4 female and 4 male crabs per tank (12 females and 12 males per experimental group); and (ii) whole ovary from 4 female crabs per tank (12 females per experimental group).

The total lipid content of both diets were extracted from breast muscle and ovary tissues according to Folch et al. [58]. The fatty acids in the lipid extract were trans-esterified to fatty acids methyl esters (FAME) using a base-catalyzed trans-esterification [59]. The fatty acid composition was determined by gas chromatography using a Varian GC 430 gas chromatograph (Varian Inc., Palo Alto, CA, USA), equipped with a flame ionization detector and a Supelco Omegawax™ 320 m capillary column (Supelco, Bellefonte, PA, USA). The chromatograms were recorded with the Galaxie Chromatography Data System 1.9.302.952 (Varian Inc., Palo Alto, CA, USA). Fatty acids were identified by comparing the FAME retention time with those of the Supelco 37 component FAME mix standard (Supelco, Bellefonte, PA, USA) and quantified through calibration curves, using tricosanoic acid (C23:0) (Supelco, Bellefonte, PA, USA) as an internal standard.

Breast muscle and ovary cholesterol content was determined using gas chromatography. Briefly, 0.2 mL of lipid extract was spiked with 0.5 mL of 5α-cholestane (0.2 mg/mL in chloroform; Supelco, Bellefonte, PA, USA) as an internal standard. After evaporating the solvent, 5 mL of 0.5 M KOH in methanol was added, and the mixture was heated in a water bath at 95 °C for 40 min to promote lipid saponification. Once cooled, 4 mL of distilled water and 2 mL of n-hexane were added. The upper organic phase was then carefully transferred into a vial for GC analysis. The analysis was performed using a Varian GC 430 gas chromatograph (Varian Inc., Palo Alto, CA, USA), equipped with a flame ionization detector (FID) and a Supelco SAC^TM^ fused silica capillary column (30 m × 0.25 mm i.d., 0.25-μm film; Supelco, Bellefonte, PA, USA). A sample of 1 μL was injected with a 1:100 split ratio at 300 °C. The oven temperature was programmed to increase from 130 to 290 °C at a rate of 20 °C/min over 8 min, followed by a hold at 290 °C for 11 min. The detector was maintained at 300 °C, and helium was used as the carrier gas with a constant flow rate of 1.3 mL/min.

### 2.5. Histological Analysis

Samples of the hepatopancreas and ovary from 10 female crabs per tank (30 females per experimental group) and samples of the hepatopancreas and medial vasa deferens of testis from 10 male crabs per tank (30 males per experimental group) were collected. Samples were fixed for 24 h at 4 °C (Bouin’s solution; Merck KGaA, Darmstadt, Germany) and then included in paraffin (Bio Optica, Milan, Italy) to obtain 5 μm sections following the procedure described in Randazzo et al. [60].

For each sample, three transverse sections were collected (at 200 μm intervals from each other) for the histological evaluations and stained with Mayer’s hematoxylin and eosin Y (Merck KGaA).

For the hepatopancreas, histological analyses were focused on the middle portion of the hepatopancreas tubule due to the presence of the mature stage of R cells (the main site of lipid absorption and storage in Decapoda), considered a key indicator of the nutritional condition in crustaceans [52,61]. The degree of vacuolization (lipid droplets) in R cells and the tubule diameter were measured on 20 randomly selected round-shaped tubules (with an average diameter range between 350 and 450 µm) per section (3 sections per crab, 30 females and 30 males per dietary treatment), according to Zarantoniello et al. [62].

For the gonads, 3 sections per crab (30 female and 30 male specimens per dietary treatment) were analyzed to determine: (i) the developmental stage of the oocytes and the eventual presence of atretic ones (characterized by a lack of structural integrity) in the ovary; and (ii) the storage of mature spermatophores in the lumen of the medial vasa deferens of testis [63].

Images were acquired using a combined color digital camera (Axiocam 105; Zeiss, Oberkochen, Germany) and analyzed with ZEN 2.3 software (Zeiss).

### 2.6. Molecular Analysis

The relative mRNA abundances of target genes in the hepatopancreas and pericardial organ samples from 10 male and 10 female crabs from each tank (30 males and 30 females per dietary group) were measured using real-time PCR. Total RNA extraction, cDNA synthesis, and PCR were conducted following the procedure described in Cattaneo et al. [64]. In each PCR run, the melting curve revealed one single peak, confirming the specificity of the amplification products; no peaks were detected for the two no-template controls added in each run.

The gene expression relative quantification of the ecdysone receptor (*ecr*), retinoid-X receptor (*rxr*), vitellogenin (*vtg*, only in female crabs), and heat shock protein 90 (*hsp90*) was performed in hepatopancreas samples. Relative quantification of the crustacean hyperglycemic hormone (*chh*) was performed in pericardial organ samples. The primer sequences are summarized in Table 2. The mRNA levels of target genes analyzed were calculated through the software iQ5 optical system version 2.0 (Bio-Rad, Hercules, CA, USA) and the GeneEx Macro iQ5 Conversion and GeneEx Macro iQ5 files, using the expression of two reference genes (18S ribosomal protein, *18s*; beta-actin, *β-actin*) in both tissues.

### 2.7. Statistical Analysis

The tanks were used as the experimental unit for data related to survival rate and biometric measurements, while crabs were considered the experimental unit for all remaining analyses. All data were checked for normality (Shapiro–Wilk test) and homoscedasticity (Levene’s test). Data related to biometric measurements, histological and gene expression analysis, as well as lipid content and fatty acid profile of breast muscles and the ovary were analyzed using a one-way ANOVA followed by a Tukey’s multiple comparison test. Significance was set at *p* < 0.05.

The statistical software package Prism-8 (GraphPad Software, San Diego, CA, USA) was used for the data analysis of the results of biometric, histological, and gene expression measurements, while the statistical software package SAS (2021) was utilized to analyze the lipid content and fatty acid data.

## 3. Results

### 3.1. Survival and Biometric Measurements

The survival rate and biometric measurements are reported in Table 3. The survival rate and carapace length and width did not significantly vary among the experimental groups in male or female crabs.

Considering males, specimens from the Ter group were characterized by a significantly (*p* < 0.05) lower final body weight and weight gain compared to the other experimental groups, while no significant differences were detected in terms of HSI or GSI. As regards the females, crabs from the Ter group showed a significantly lower final body weight and weight gain and significantly higher HSI and GSI values compared to the other experimental groups.

### 3.2. Lipid Content and Fatty Acid Profile of Breast Muscles and Ovaries

The total lipids and fatty acid profiles of breast muscle of blue crab males and females are presented in Table 4 and Table 5, respectively. Regarding the total lipid and cholesterol content in breast muscles of both males and females, no significant differences were evident among the experimental groups.

Considering the fatty acid profiles of breast muscle of males, palmitic (C16:0) and stearic (C18:0) acids were the most represented saturated fatty acids (SFA) and showed an opposite trend, with a significant decrease and increase, respectively, from the Mar to Ter groups. However, the total SFA content was not significantly different among the experimental groups. Considering monounsaturated fatty acids (MUFA), the most represented fatty acid was oleic acid (C18:1n9), which was significantly higher in the Ter group compared to the other ones that did not evidence significant differences between them. The total amount of MUFA was significantly lower in the Mix group compared to the other ones. Finally, as regards PUFA, the Ter group was characterized by significantly lower EPA and DHA percentages and, accordingly, a significantly lower n-3 PUFA total incidence compared to the other experimental groups. Conversely, a significantly increasing trend in linoleic (C18:2n-6; LNA) and ARA (C20:4n-6) percentages and, accordingly, in the n-6 PUFA total amount was evidenced from the Mar to Ter groups.

As regards the fatty acid profile of breast muscle of females (Table 5), the most represented SFA were: (i) palmitic acid (C16:0), which was not significantly different among groups; and (ii) stearic acid (C18:0), which was significantly higher in the Ter group compared to the Mar one. No significant differences were detected among experimental groups in terms of total SFA. Considering MUFA, no significant differences were evident in terms of oleic acid (C18:1n9; the most represented fatty acid) and MUFA total percentage among the experimental groups. As regards PUFA, the Ter group was characterized by a significantly lower DHA incidence and, accordingly, a significantly lower n-3 PUFA total percentage compared to the other experimental groups. No significant differences were evident in terms of EPA content. In contrast, a significantly increasing trend in LNA content and, accordingly, in n-6 PUFA total percentage was evidenced from the Mar to Ter groups, even if milder compared to that observed for males (Table 4). The ARA incidence was significantly higher in both Mix and Ter groups compared to Mar.

Considering the lipid content and the fatty acid profile of the ovary (Table 6), similar trends compared to those described for females’ breast muscles was obtained, except for: (i) a significantly lower SFA total amount in the Mix group compared to the other experimental groups; and (ii) a significantly lower EPA content in the Ter group compared to both the Mar and Mix ones.

### 3.3. Histology of the Hepatopancreas and Gonads

Both female and male crabs from all experimental groups showed a normal structure of all tissues analyzed. Furthermore, the same structural features of the hepatopancreas and ovary or medial vasa deferens were evident among the experimental groups. 

**Figure 1:**
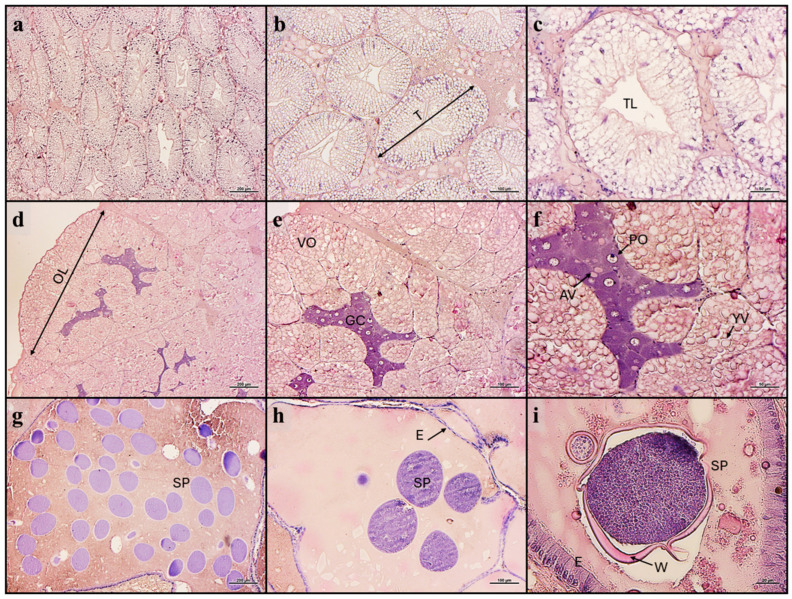
Example of histomorphology of (**a**–**c**) hepatopancreas, (**d**–**f**) ovary, and (**g**–**i**) medial vasa deferens of testis of blue crabs fed the experimental diets. Scale bars: (**a**,**d**,**g**) 200 µm; (**b**,**e**,**h**) 100 µm; (**c**,**f**) 50 µm; (**i**) 20 µm. Abbreviations: T, tubule diameter; TL, tubular lumen; OL, ovarian lobe; VO, vitellogenic oocyte; GC, germinal center; PO, primary oocyte; AV, acidophilic vesicle; YV, yolk vesicle; SP, spermatophores; E, epithelium; W, glyco-proteinaceous wall.

Considering the hepatopancreas (Figure 1a–c), no alterations in the tissue architecture were evident in male or female crabs that showed a tubular structure characterized by diffused R cells with highly abundant lipid droplets.

As regards the ovary (Figure 1d–f), female crabs from all experimental groups were sexually mature at the same gonadal stage. Particularly, all ovaries were organized in lobes, which presented, in their central region, the germinative center occupied by primary oocytes (pre-vitellogenic), with a strong basophilic cytoplasm and large acidophilic vesicles. The outer region of each lobe was occupied by vitellogenic oocytes (early to mid-vitellogenesis) filled with numerous yolk granules, which occupied most of the cytoplasm and obstructed nucleus observation. The pressure of vitellogenic oocytes against the connective capsule led to the irregular shape of these cells. In each sample, no phenomena of atresia were evident. 

Finally, male crabs from all the experimental groups were sexually mature and characterized by the storage of mature spermatophores in the medial vas deferens. Particularly, the vas epithelial cells were flattened by the intense secretion in the lumen, which was filled with mature spermatophores and granular secretions immersed in a homogeneous matrix constituting the seminal fluid (Figure 1g–i).

### 3.4. Real-Time PCR Results

**Figure 2:**
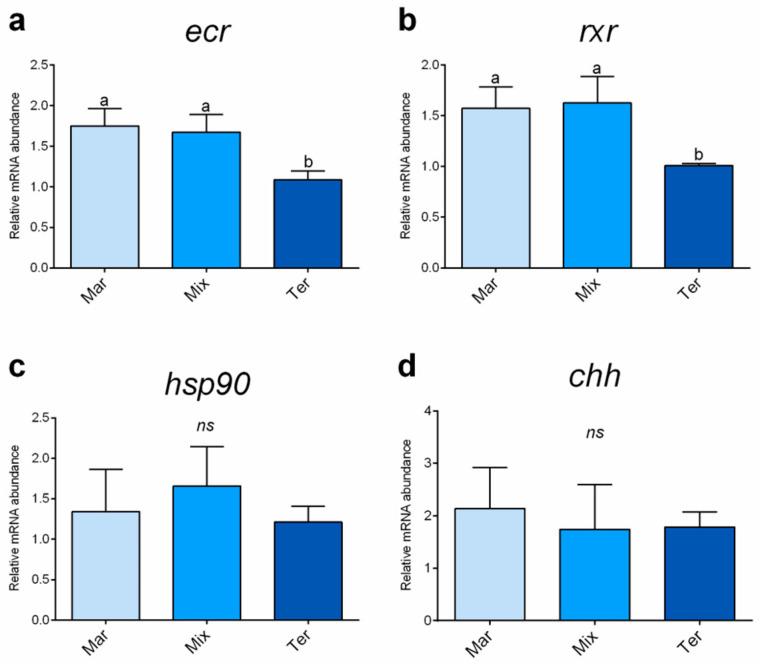
Relative mRNA abundance of (**a**) *ecr*, (**b**) *rxr*, and (**c**) *hsp90* analyzed in the hepatopancreas and (**d**) *chh* analyzed in the pericardial organ of blue crab males from the different dietary treatments. Values are presented as mean ± SD (*n* = 5). ^a,b^ different letters denote statistically significant differences among the experimental groups. *ns*, no significant differences (*p* > 0.05).

As regards blue crab males, a significantly (*p* < 0.05) lower relative *ecr* and *rxr* expression was observed in the Ter group compared to the Mar and Mix ones, which did not show significant differences (Figure 2a,b). No significant differences were detected among the experimental groups in terms of the relative expression of both *hsp90* and *chh* (Figure 2c,d).

**Figure 3:**
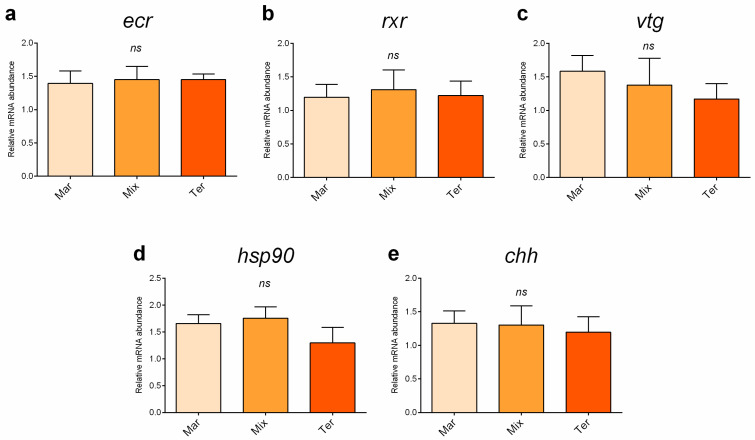
Relative mRNA abundance of (**a**) *ecr*, (**b**) *rxr*, (**c**) *vtg*, and (**d**) *hsp90* analyzed in the hepatopancreas and (**e**) *chh* analyzed in the pericardial organ of blue crab females from the different dietary treatments. Values are presented as the mean ± SD (*n* = 5). *ns*, no significant differences (*p* > 0.05).

Considering blue crab females, no significant differences in the relative expression of *ecr*, *rxr*, *vtg*, *hsp90*, or *chh* were evident among the experimental groups (Figure 3).

## 4. Discussion

In decapod crustaceans, the availability of proper feed sources can deeply affect survival, quality traits, fecundity, and overall welfare in both wild and controlled conditions [42,65]. For example, it has been demonstrated that the provision of seaweed (*Ulva lactuca*) to blue crabs over a 12-week feeding trial led to higher mortality, a reduction in the energy reserves with consequences on reproductive performance, and the occurrence of aggressive behavior [66]. Furthermore, dietary lipids and, particularly, long-chain PUFA have been demonstrated to be crucial for many basic functions in crabs, including growth, reproduction, and maintenance of the structural integrity of tissues [46,48,67].

The different dietary treatments tested in the present study did not significantly affect survival (higher than 90%) and the overall health status of both male and female blue crabs, as confirmed by the absence of pathological alterations in the architecture of all tissues analyzed and by the expression of stress markers (*hsp90* and *chh*). In addition, the absence of significant differences in both survival and stress responses confirmed proper maintenance of the blue crabs throughout the whole trial since crustaceans are usually susceptible to the water quality in controlled conditions [68]. In decapods, the stress response to environmental factors is mainly governed by the crustacean hyperglycemic hormones (CHH) [69,70,71], first identified in the medulla terminalis X-organ and sinus gland in the eyestalk [72,73] and more recently found in different tissues, including the pericardial organ [74,75,76]. The physiological mechanism governed by the CHH is closely related to nutrient availability. In fact, the proper stress response is represented by the possibility to achieve a hyperglycemic status through the mobilization of carbohydrates and lipids from the hepatopancreas reserves [71,77]. In the present study, all crabs analyzed were characterized by proper nutrient storage in the hepatopancreas, as evidenced by the same degree of R cells abundance and lipid vacuolization, highlighting the proper welfare status of crabs and eventual ability to fully sustain potential stressful conditions.

Besides the stress response, nutrient availability and lipid metabolism are crucial to sustain other energy-demanding processes, including growth, molting, and reproduction [78,79,80]. Particularly, lipid accumulation in the muscle and hepatopancreas is considered a crucial factor to evaluate the nutritional intake of crabs [81].

In the present study, in both male and female crabs, the lipid content and the fatty acid profiles of breast muscle reflected the dietary profile. This result is in accord with previous studies in crustaceans that evidenced a strong correlation between the diet and the chemical nutritional composition of the muscles [82,83]. The absence of differences among the experimental groups in terms of SFA (mainly represented by palmitic and stearic acids) and MUFA (except for a slightly lower value in Mix group) provided important information about the nutritional status of this tissue [81]. In fact, the β-oxidation of SFA, particularly of palmitic and stearic acids, constitute the main source of energy for the maintenance of neuromuscular functions in crabs [84]. Furthermore, previous studies conducted on mud crab (*Scylla serrata*) and Chinese mitten crab (*Eriocheir sinensis*) evidenced an increase in muscle MUFA content in response to a diet deficient in essential fatty acids [85]. In the present study, both males and females of the Ter group showed comparable values of MUFA in muscle to those observed in Mar and Mix ones. This result can be interpreted as a proper provision of essential fatty acids (both n-3 and n-6 long-chain PUFA [46]), at least to meet the minimal requirements for sustaining the overall welfare and reproductive status but possibly at the expense of growth. In fact, both males and females from the Mix group always showed comparable results compared to those observed in the Mar one, indicating how blue crabs can rapidly recover (20 days in the present study) from a period of suboptimal nutrition, especially in terms of n-3 PUFA. Conversely, both male and female blue crabs from the Ter group were characterized by a lower final body weight compared to that observed for the Mar and Mix ones. Long-chain PUFA are involved in crustacean growth, regulating molting and serving as precursors of the eicosanoids necessary for this process [86]. Despite the important role of both n-3 and n-6 long-chain PUFA, growth performances are properly sustained by high levels of dietary EPA and DHA, as also previously demonstrated in the swimming crab (*Portunus trituberculatus*) [87]. For this reason, in the present study, the 60-day provision of a diet particularly rich in n-6 PUFA and low in n-3 PUFA, probably did not provide sufficient levels of EPA and DHA to simultaneously support growth and reproductive status in both male and female blue crabs. In fact, all crabs used in the present study were in the intermolt phase and sexually mature, as firstly evidenced by the bright orange coloration of the ovary and by the presence of a pale pink secretion that filled the medial vas deference at sampling time [53,88], with this result also confirmed by the histological analyses. Reproduction in crabs is an energetically expensive process, with lipids being the main source of energy [89]. In adult specimens, high levels of EPA, DHA, and ARA are in fact necessary for gonad maturation [90].

In crabs, the main site of lipid storage required to sustain reproduction is represented by the hepatopancreas [43,91]. This organ is involved in the nutrients’ gradual transfer to the gonads, assisting their development [92,93]. In the present study, all the specimens analyzed were characterized by a proper nutritional status, as evidenced by the same storage degree of dietary lipids in the hepatopancreas. Accordingly, both male and female crabs from all experimental groups highlighted the proper development of the reproductive system, as supported by the HSI and GSI values. Particularly, in the case of the Ter group, both males and females showed higher levels of these indexes compared to the other experimental groups, indicating the preservation of the hepatopancreas and the gonads to sustain reproduction at the expense of growth. In fact, the size of the crab hepatopancreas is an indicator of nutrient accumulation [94], whilst gonadal mass reflects the maturation stage in both females and males [53,88].

Considering female crabs, oogenesis generally consists of a proliferative phase (oogonia multiplication) and a growth phase (previtellogenic and vitellogenic stages) characterized by the differentiation and maturation of the oocytes through the accumulation of yolk [57]. In the present study, the ovary of all specimens from the different experimental groups were found in early to mid-secondary growth (vitellogenesis), as evidenced by the well-defined germinal zone (that progressively become less evident during development) and by the presence, in the outer regions, of oocytes filled with numerous yolk granules, which represent the main form of nutrient storage inside the cell. The absence of yolk vesicles coalescence or atresia phenomena further supported that females were in a phase of ovarian development that still involved the accumulation of abundant nutrient reserves. For this reason, it can be stated that the provision of all dietary treatments allowed proper nutrient storage in the hepatopancreas, which, in turn, sustained sufficient nutrient transfer to the developing oocytes, ensuring proper gonadal development in female blue crabs. This result was also supported by the expression of *ecr*, *rxr*, and *vtg*, which did not show significant differences among the experimental groups. In crustaceans, the ecdysone receptor (EcR) and RXR are involved, among other functions, in the positive regulation of vitellogenin synthesis in the hepatopancreas, forming a heterodimer and initiating a transcriptional cascade of ecdysteroid-responsive genes in target tissues [44,45]. Vitellogenin is exclusively synthesized in the hepatopancreas and translocated as a high-density lipoprotein to the ovary, where it is internalized in the oocytes via endocytosis mediated by vitellogenin receptors [95,96,97]. Vitellogenin represents the major protein that accumulates within the ovary during this process [98]. However, during the vitellogenic stage, lipids are fundamental for the ovarian maturation process and represent important components for the yolk [57,97]. In fact, MUFA as well as n-3 and n-6 long-chain PUFA progressively accumulate during ovarian maturation, acting as energy reservoirs and essential components for ensuring reproductive success in crabs [90,97,99]. In the present study, the fatty acid profiles of the ovary samples reflected that of the experimental diets. While both the Mar and Mix groups were characterized by higher levels of n-3 PUFA compared to the n-6 ones, the Ter group showed an opposite trend. These results align with findings obtained in other crab species. In fact, orange mud crab (*Scylla olivacea*) females fed a fish-based diet (e.g., *Decapterus* spp.) exhibited higher levels of both PUFA and MUFA compared to SFA, reflecting nutritional changes associated with gonad maturation [100]. Additionally, Dvoretsky et al. [101] analyzed the fatty acid profiles of ovaries from female red king crab (*Paralithodes camtschaticus*), harvested from their natural environment, obtaining consistent outcomes. In the present study, the need for both n-3 and n-6 long-chain PUFA to sustain proper ovarian development was potentially satisfied in all experimental groups, as confirmed by the histological analyses. In fact, it has been demonstrated that, independent of the dietary fatty acid profile, DHA and EPA are preferentially accumulated in the ovary in the reproductive phase due to their crucial role for crab embryogenesis [50]. This is consistent with the hypothesis that female crabs, challenged for 60 days with a diet of terrestrial origin rich in n-6 PUFA and low in n-3 PUFA, preferentially sustained reproduction at the expense of growth.

Despite the lower energetic investment for gonad development and gamete maturation compared to females, a similar scenario was detected in male crabs. The development of the male reproductive system is characterized by an increase in the volume of the vas deferens and may be associated with the production of seminal fluid, which acts in the transfer of sperm and the formation of the sperm plug in the seminal receptacles of females [53]. In the present study, blue crab males from all the experimental groups were characterized by the storage of mature spermatophores in the medial vas deferens, included in the granular eosinophilic seminal fluid and, accordingly, by comparable GSI values. However, male specimens from the Ter group evidenced a lower final body weight, further supporting that the long-term provision of a diet poor in n-3 PUFA preserved gonadal maturation, compromising growth. Accordingly, *ecr* and *rxr* expression was lower in male crabs from the Ter group, being signals involved (besides reproduction) in molting, metamorphosis, and growth [45,102]. In fact, in crustaceans, EcR forms a functional heterodimer with RxR, which binds to ecdysone, forming an active trimer. This complex regulates growth and molting functions by activating target genes [44,81]. This result is in accordance with a previous study that demonstrated the positive correlation between growth performance and *ecr* and *rxr* expression in Chinese mitten crab (*Eriocheir sinensis*) [103].

## 5. Conclusions

Blue crabs prioritize reproductive investment rather than growth by directing vital resources to their reproductive organs when subjected to suboptimal diets. This intriguing finding has implications for species survival: when feed supply or quality diminishes, the blue crab utilizes its biological reserves to enhance reproduction. By generating a large number of larvae, the chances of establishing new populations in areas with suitable feed ensures the population’s survival. In conclusion, this preliminary study provides important insights on how diet composition, especially in terms of long-chain PUFA, can affect blue crab growth and reproduction, highlighting the preferential investment in the future generation in case of suboptimal nutrition. This information can also be useful in the Adriatic area to promote aquaculture practices to increase the blue crab size and thus its economic value.

## Figures and Tables

**Table 1:** Fatty acid profile (g fatty acid/100 g total fatty acid methyl esters) of marine (sardinella fillet) and terrestrial (bovine heart) diets used in the present study.

	Marine Diet	Terrestrial Diet
**Total lipids**	2.83 ± 1.56	3.52 ± 0.58
**Fatty acids**		
C14:0	3.16 ± 0.25	2.74 ± 0.90
C16:0	24.62 ± 0.77	18.51 ± 2.34
C16:1n-7	8.16 ± 0.06	1.12 ± 0.02
C18:0	8.65 ± 0.15	16.04 ± 0.62
C18:1n-9	21.91 ± 3.61	21.78 ± 1.87
C18:1n-7	3.90 ± 0.22	3.12 ± 0.07
C18:2n-6; LNA	0.89 ± 0.38	24.13 ± 3.26
C20:4n-6, ARA	2.63 ± 0.50	7.48 ± 1.93
C20:5n-3, EPA	5.10 ± 0.33	N.D.
C22:5n-3	1.72 ± 0.13	0.36 ± 0.12
C24:0	0.12 ± 0.06	0.16 ± 0.05
C22:6n-3, DHA	11.27 ± 2.86	0.27 ± 0.08
ΣSFA	39.33 ± 0.57	39.33 ± 4.15
ΣMUFA	35.97 ± 3.94	26.85 ± 1.68
Σn-3 PUFA	18.85 ± 3.28	1.19 ± 0.21
Σn-6 PUFA	5.19 ± 1.23	32.55 ± 5.59

Data are expressed as mean ± SD (*n* = 3). For calculating the *Σ* classes, 48 fatty acids were used, but those with a quantity below 1% of total fatty acid methyl esters (FAME) are not listed in the table. Abbreviations: SFA, saturated fatty acids; MUFA, monounsaturated fatty acids; PUFA, polyunsaturated fatty acids; N.D., not detected.

**Table 2:** Forward and reverse sequences and NCBI IDs of primers used in the present study.

Genes	Forward Sequence (5′-3′)	Reverse Sequence (5′-3′)	NCBI ID
*ecr*	GCATTGTGTTTGGAAATACCTTGCC	GCCCTCAATGCATCGAGGTATATTT	HQ630857
*rxr*	CACCATCGACAAGAGACAGAGGAA	AGATAGCGCCACAGGAGGACTCT	HQ630860
*vtg*	TGTACAGCTGAAAGGCGTGG	CATGGGCCGAGAACAGTCA	DQ314748
*hsp90*	CACCGACAACATCAAGCTGTAC	ACACCACGCACAAAGTTGAG	DQ667139
*chh*	CTGTATGATGGCCACGCTCTCA	CAGCTCGTTGAAGATGGCTCTGT	AY536012
*18s (hk)*	TCAAGTGTCTGCCTTATCAGCT	TCGGATGAGTCTCGCATCGT	AY743951.1
*β-actin (hk)*	TCGAGCACGGTATTGTCACC	GTACATGGCGGGAGTGTTGA	DQ084066.1

Abbreviations: *hk*, housekeeping gene.

**Table 3:** Biometric measurements of blue crab fed the experimental diets.

	Mar	Mix	Ter	*p*-Value
		**Males**		
SR (%)	95.1 ± 2.7	91.6 ± 1.3	92.3 ± 2.8	0.2388
CL (cm)	6.2 ± 0.6	5.8 ± 0.3	5.9 ± 0.3	0.362
CW (cm)	13.3 ± 0.6	13.2 ± 0.7	12.8 ±1.3	0.764
FBW (g)	176.8 ± 20.7 ^a^	164.7 ± 6.5 ^a^	107.0 ± 14.7 ^b^	0.002
WG (g)	88.8 ± 20.3 ^a^	80.1 ± 8.2 ^a^	25.3 ± 17.3 ^b^	0.0112
HSI (%)	8.1 ± 0.3	8.1 ± 0.5	7.8 ± 2.5	0.959
GSI (%)	1.6 ± 0.5	1.6 ± 0.7	2.3 ± 1.0	0.462
		**Females**		
SR (%)	91.1 ± 3.3	90.8 ± 1.6	88.8 ± 2.7	0.5425
CL (cm)	5.8 ± 0.3	5.5 ± 0.3	5.4 ± 0.1	0.720
CW (cm)	12.4 ± 0.9	11.6 ± 0.7	11.4 ± 1.2	0.472
FBW (g)	156.0 ± 14.1 ^a^	145.7 ± 18.8 ^a^	87.7 ± 8.0 ^b^	0.002
WG (g)	76.7 ± 25.7 ^a^	60.7 ± 20.0 ^a^	9.0 ± 4.6 ^b^	0.0043
HSI (%)	8.9 ± 1.3 ^b^	8.5 ± 1.0 ^b^	14.1 ± 1.1 ^a^	0.001
GSI (%)	4.8 ± 0.8 ^b^	5.3 ± 1.0 ^b^	7.9 ± 0.8 ^a^	0.009

Data are expressed as mean ± SD (*n* = 3). ^a,b^ Within each line, different letters denote statistically significant differences among the experimental groups. Abbreviations: SR, survival rate; CL, carapace length; CW, carapace width; FBW, final body weight; WG, weight gain; HSI, hepatosomatic index; GSI, gonadosomatic index.

**Table 4:** Total lipids (g/100 g fresh tissue), cholesterol (mg/100 g fresh tissue), and fatty acid profiles (% of total FAME) of breast muscle samples from blue crab males fed the experimental diets.

	Mar	Mix	Ter	RMSE	*p*-Value
**Total lipids**	1.25	1.04	1.60	0.477	ns
**Cholesterol**	39.54	35.90	50.20	10.472	ns
**Fatty acids**					
C14:0	2.16 ^a^	0.96 ^b^	1.28 ^b^	0.342	0.0007
C16:0	20.18 ^a^	18.03 ^b^	17.63 ^b^	1.123	0.01
C16:1n-7	4.80 ^a^	4.33 ^ab^	3.60 ^b^	0.057	0.04
C18:0	7.59 ^c^	10.07 ^b^	11.80 ^a^	0.917	0.0002
C18:1n-9	18.39 ^b^	16.55 ^b^	20.80 ^a^	1.369	0.0002
C18:1n-7	2.80	2.79	2.86	0.269	ns
C18:2n-6, LNA	2.36 ^c^	6.26 ^b^	14.24 ^a^	1.629	<0.0001
C20:1n-9	1.04 ^a^	0.37 ^b^	0.36 ^b^	0.113	<0.0001
C20:4n-6, ARA	3.42 ^c^	5.96 ^b^	7.77 ^a^	0.462	<0.0001
C20:5n-3, EPA	10.19 ^a^	12.70 ^a^	6.68 ^b^	1.932	0.007
C22:6n-3, DHA	18.51 ^a^	15.15 ^b^	5.83 ^c^	1.890	<0.0001
∑SFA	32.71	31.78	34.09	1.364	ns
∑MUFA	29.08 ^a^	24.53 ^b^	28.16 ^a^	1.836	0.01
∑n-3 PUFA	30.65 ^a^	29.68 ^a^	13.66 ^b^	3.346	<0.0001
∑n-6 PUFA	6.85 ^c^	13.38 ^b^	23.52 ^a^	1.715	<0.0001

Data are expressed as mean coupled with root mean square error (RMSE; *n* = 12). For calculating the *Σ* classes, 44 fatty acids were used, but those below 1% of total FAME are not listed in the table. ^a–c^ different letters denote statistically significant differences among the experimental groups within the same row. Abbreviations: SFA, saturated fatty acids; MUFA, monounsaturated fatty acids; PUFA, polyunsaturated fatty acids; ns, no significant differences (*p* > 0.05).

**Table 5:** Total lipids (g/100 g fresh tissue), cholesterol (mg/100 g fresh tissue), and fatty acid profiles (% of total FAME) of breast muscle samples from blue crab females fed the experimental diets.

	Mar	Mix	Ter	RMSE	*p*-Value
**Total lipids**	1.17	1.15	1.06	0.13	ns
**Cholesterol**	35.56	37.03	33.12	4.60	ns
**Fatty acids**					
C14:0	1.68	1.22	1.09	0.36	ns
C16:0	19.90	18.13	17.55	1.13	ns
C16:1n-7	4.71	5.03	5.21	1.35	ns
C18:0	7.86 ^b^	9.35 ^ab^	10.64 ^a^	0.95	0.02
C18:1n-9	20.04	19.36	19.95	1.43	ns
C18:1n-7	2.93	2.95	2.66	0.26	ns
C18:2n-6, LNA	2.39 ^c^	6.08 ^b^	10.67 ^a^	0.51	<0.0001
C20:1n-9	1.02 ^a^	0.54 ^b^	0.30 ^b^	0.21	0.01
C20:4n-6, ARA	2.98 ^b^	5.74 ^a^	6.33 ^a^	0.59	0.0003
C20:5n-3, EPA	10.34	10.72	10.28	1.68	ns
C22:6n-3, DHA	18.83 ^a^	13.90 ^b^	8.22 ^c^	2.32	0.002
∑SFA	31.88	31.53	32.28	1.27	ns
∑MUFA	30.51	28.50	28.64	2.60	ns
∑n-3 PUFA	30.71 ^a^	26.17 ^a^	20.12 ^b^	3.30	0.01
∑n-6 PUFA	6.43 ^c^	13.33 ^b^	18.37 ^a^	1.13	<0.0001

Data are expressed as mean coupled with root mean square error (RMSE; *n* = 12). For calculating the *Σ* classes, 44 fatty acids were used, but those below 1% of total FAME are not listed in the table. ^a–c^ different letters denote statistically significant differences among the experimental groups within the same row. Abbreviations: SFA, saturated fatty acids; MUFA, monounsaturated fatty acids; PUFA, polyunsaturated fatty acids; ns, no significant differences (*p* > 0.05).

**Table 6:** Total lipids (g/100 g fresh tissue) and fatty acid profiles (% of total FAME) of ovary samples from blue crab females fed the experimental diets.

	Mar	Mix	Ter	RMSE	*p*-Value
**Total lipids**	9.21	9.72	5.27	2.68	ns
**Fatty acids**					
C14:0	3.29 ^a^	2.43 ^b^	1.61 ^c^	0.22	0.01
C16:0	23.45	21.61	20.58	0.70	ns
C16:1n-7	8.48	6.22	6.95	2.74	ns
C18:0	6.67 ^c^	7.17 ^b^	10.96 ^a^	0.21	0.001
C18:1n-9	18.86	20.38	19.71	1.94	ns
C18:1n-7	2.94 ^a^	3.45 ^a^	2.49 ^b^	0.19	0.04
C18:2n-6; LNA	2.10 ^c^	5.45 ^b^	11.58 ^a^	1.02	0.01
C20:4n-6; ARA	2.38 ^c^	3.71 ^b^	7.88 ^a^	0.19	0.0002
C20:5n-3; EPA	7.47 ^a^	6.58 ^a^	3.91 ^b^	0.69	0.03
C22:6n-3; DHA	15.28 ^a^	15.04 ^a^	5.20 ^b^	1.64	0.01
∑SFA	36.51 ^a^	33.93 ^b^	36.99 ^a^	0.47	0.01
∑MUFA	32.91	31.70	30.82	1.62	ns
∑n-3 PUFA	24.72 ^a^	23.51 ^a^	10.75 ^b^	1.15	0.002
∑n-6 PUFA	5.55 ^c^	10.60 ^b^	21.18 ^a^	0.71	0.001

Data are expressed as mean coupled with root mean square error (RMSE; *n* = 12). For calculating the *Σ* classes, 45 fatty acids were used, but those below 1% of total FAME are not listed in the table. ^a–c^ different letters denote statistically significant differences among the experimental groups within the same row. Abbreviations: SFA, saturated fatty acids; MUFA, monounsaturated fatty acids; PUFA, polyunsaturated fatty acids; ns, no significant differences (*p* > 0.05).

## Data Availability

The original contributions presented in this study are included in the article. Further inquiries can be directed to the corresponding authors.
